# Prevalence status and associated factors of wrist postural injury in the Chinese occupational population

**DOI:** 10.3389/fpubh.2022.1047814

**Published:** 2022-11-23

**Authors:** Nengzhou Chen, Guanlin Li, Xin Sun, Meibian Zhang, Huadong Zhang, Ruijie Ling, Yiming Liu, Gang Li, Zaoliang Ren, Yan Yin, Hua Shao, Hengdong Zhang, Jiajie Li, Bing Qiu, Dayu Wang, Qiang Zeng, Zhanhui Liang, Rugang Wang, Jianchao Chen, Danying Zhang, Liangying Mei, Yongquan Liu, Jixiang Liu, Chengyun Zhang, Tianlai Li, Zhongxu Wang, Qingsong Chen, Ning Jia

**Affiliations:** ^1^Guangdong Public Health Testing and Evaluation Engineering Technology Center, Guangdong Pharmaceutical University, Guangzhou, China; ^2^National Institute of Occupational Health and Poisoning Control, Chinese Center for Disease Control and Prevention, Beijing, China; ^3^Chongqing Center for Disease Control and Prevention, Chongqing, China; ^4^Xinhua Hospital, Hubei, China; ^5^Guangzhou Occupational Disease Prevention and Treatment Hospital, Guangzhou, China; ^6^Liaoning Provincial Health Service Center, Shenyang, China; ^7^Guizhou Province Hospital for Occupational Disease Prevention and Treatment, Guiyang, China; ^8^Shanghai Center for Disease Control and Prevention, Shanghai, China; ^9^Shandong Academy of Occupational Health and Occupational Disease Control, Jinan, China; ^10^Jiangsu Provincial Center for Disease Control and Prevention, Nanjing, China; ^11^Civil Aviation Medical Center, Civil Aviation Administration of China, Beijing, China; ^12^Tianjin Occupational Disease Prevention and Treatment Hospital, Tianjin, China; ^13^Tianjin Center for Disease Control and Prevention, Tianjin, China; ^14^Beijing Center for Disease Control and Prevention, Beijing, China; ^15^Fujian Provincial Center for Occupational Disease and Chemical Poisoning Prevention and Control, Fuzhou, China; ^16^Guangdong Provincial Occupational Disease Prevention and Control Institute, Guangzhou, China; ^17^Hubei Provincial Center for Disease Control and Prevention, Wuhan, China; ^18^Institute of Occupational Medicine of Jiangxi, Nanchang, China; ^19^Ningxia Center for Disease Control and Prevention, Yinchuan, China; ^20^Sichuan Provincial Center for Disease Control and Prevention, Chengdu, China; ^21^Shanxi Provincial Center for Disease Control and Prevention, Xian, China

**Keywords:** Chinese worker, wrist posture, wrist injury, damage, associated factors

## Abstract

**Objective:**

This study investigated the prevalence of wrist injuries in 15 industries and different types of work in China. Study on the associated factors of wrist injuries provides a scientific basis for prevention and treatment of wrist diseases in occupational workers.

**Methods:**

A cross-sectional study of musculoskeletal symptoms of related practitioners in 15 industries, including automobile manufacturing, was conducted to retrieve worker demographic information, working wrist posture, and pain conditions. Multivariable binary logistic regression analyses were performed to identify factors associated with work-related musculoskeletal disorders (WMSDs).

**Results:**

The prevalence of wrist injuries among the study population was 13.2%. Toy manufacturing, animal husbandry, automobile manufacturing, shoe manufacturing, and biopharmaceutical manufacturing had the highest wrist injury rates at 29.1, 19.1, 14.9, 14.9, and 14.0%, respectively. Among the types of jobs, enamel workers (63.0%), butchers (43.6%), combers (32.5%), welders (31.3%), and scaffolders (26.5%) had the highest prevalence rates. Based on the final multivariate logistic regression analysis: female [odds ratios (OR) = 1.24; 95% confidence interval (CI), 1.15–1.35], 6–10 years of service (OR = 1.11; 95% CI, 1.03–1.18), >10 years of service (OR = 1.15; 95% CI, 1.06–1.25), frequent upward and downward flexion in wrist posture at work (OR = 1.81; 95% CI, 1.84–2.11), and frequent wrist placement on the edge of angular objects increased the OR of injury (OR = 1.52; 95% CI, 1.44–1.61). Need to squeeze objects tightly while working (OR = 1.72; 95% CI, 1.57–1.89), prolonged wrist flexion (OR = 1.86; 95% CI, 1.75–1.97), and work hand position above the shoulder for prolonged periods (OR = 1.11; 95% CI, 1.04–1.19) also suggested the relationship between these factors and the higher prevalence of wrist injury in the workers. The associated factor was physical activity (OR = 0.86; 95% CI, 0.80–0.94).

**Conclusion:**

This study suggested the relationship between these factors and the higher prevalence of wrist injury in the toy manufacturing, animal husbandry, automobile manufacturing, and shoe-making industries, enamel workers, butchers, and combers. And are work types that require special attention. Females, working age, physical activity, and abnormal posture of the wrist were factors significantly associated with WMSDs.

## Introduction

Work-related musculoskeletal disorders (WMSDs) are injuries or diseases of the muscles, nerves, tendons, joints, cartilage, and discs caused by work-related factors (1). Musculoskeletal pain is a widespread and expensive healthcare issue worldwide (2). Physical injuries are related to gradual damage to tissues and organs in the body (3). Additionally, WMSDs affect the body organs. The spinal vertebrae and hands are the most sensitive organs with risk of WMSDs (4). Wrist pain is a type of musculoskeletal disorder with an annual consultation prevalence of 0.058% in the UK, making it the fourth most common upper extremity pain site after the shoulders, hands, and elbows. Wrist pain is very common in people who perform daily manual labor and sports, but is less common in the general population and non-manual workers. Although wrist pain is less frequent than back, shoulder, hip, or knee pain, it makes up a significant amount of total strain on the musculoskeletal system (5, 6). Walker-Bone et al. showed that 10% of the general population have non-specific hand and wrist pain (6).

The total number of cases of work-related musculoskeletal diseases published on the UK Labor Department official website between 2021 and 2021 was 470,000, with a prevalence rate of 1,420 per 100,000 workers. Construction, human health, and social work activities have significantly higher prevalence rates than the overall industry average. Compared to all occupational groups, the prevalence of work-related musculoskeletal disorders was significantly higher among skilled workers, nurses, leisure workers, factory workers, and machine operators over a 3-year period (2018–2021). According to a 2019 survey in China, the prevalence of WMSDs in the total working population was 42.9%, with wrist pain accounting for 13.4% of the total population. The three industries with the highest prevalence of wrist diseases were toy manufacturing, animal husbandry, and biopharmaceutical manufacturing (7).

Current research indicates that age and female sex are associated factors for musculoskeletal disorders (8, 9); however, smokers and overweight people are also at increased risk of developing WMSDs (10). Lower socioeconomic status, including reductions in education, income, and occupation is associated with WMSDs (11), and work-related factors, such as night shifts, years of service (12), and physical exposure in the work environment are also associated factors for WMSDs (13).

High repetition can cause WMSDs, with or without the use of force. There are a number of different tissue types, such as bones, tendons, muscles, and loose connective tissue. Early discrete tissue injury initiates an acute inflammatory response that can be controlled by tissue healing under conditions of flow repetition and low effort or by more tissue damage. The musculoskeletal system can become overloaded by repetitive motion, bad posture, and continuous excessive force, which increases the risk of developing WMSDs (14). The wrist is linked with longest absenteeism, which means that compared to other anatomical locations, the wrist is linked to a larger loss of productivity and earnings. Epidemiological studies have associated repeated and powerful hand-intensive jobs with onset and severity of hand and wrist WMSDs. When performing such duties in conditions of awkward wrist and forearm posture, severe low temperature, or vibration, these disorders deteriorate more quickly (15). China, as an industrialized country, has a large number of occupational workers and large base of WMSDs; however, more studies are still required to understand the illness state and associated factors for local occupational groups.

Through a survey of enterprise workers in key industries in China, this study found the prevalence of musculoskeletal diseases of the wrists of occupational workers in different industries, clarified the associated factors affecting wrist injuries, and provided a scientific basis for formulating corresponding measures for occupational workers' health.

## Methods

### Research design and themes

The study runs from 2018 to June 2021.This study adopted the stratified cluster sampling method to select all workers who were on duty and met the inclusion criteria from representative enterprises in key industries (16) in seven regions: North China, East China, Central China, South China, South-West China, North-West China, and North-East China. Inclusion criteria for the study participants was working experience of more than 1 year. Exclusion criteria include the following: congenital spinal deformity, non-WMSDs due to trauma, infectious diseases, malignant tumors, etc. Selection of key industries was based on representative industries that are closely related to the occurrence of WMSDs. We selected automobile manufacturing, footwear manufacturing, biopharmaceutical manufacturing, electronic equipment manufacturing, shipbuilding and related device manufacturing, petrochemical industry, construction industry, furniture manufacturing, coal mining and washing, animal husbandry, medical personnel, automobile 4S shops, vegetable greenhouses, civil aviation crews, toy manufacturing, and other 15 industries or working groups with a total of 64,052 individuals. A total of 61,034 questionnaires were received with a questionnaire response rate of 94.6%, and 57,501 valid questionnaires were received with a questionnaire response rate of 94.2%. A one-on-one face-to-face survey was used. The significance level (α) was 0.05, the confidence level (1–α) was 0.95, the allowable error (δ) was 0.023, and the proportion (p) was 0.2324, and the calculated sample size was 1,321.The sample size calculation formula is:


$$\begin{array}{l} N={\left(\frac{Z_{1-\alpha }}{\delta }\right)}^{2}\times \text{P}\times \left(\text{1-P}\right) \end{array}$$

### Survey design

The ergonomic evaluation and analysis system for WMSDs developed by the study group, Occupational Protection and Ergonomics Research Office, Institute of Occupational Hygiene and Poison Control, Chinese Center for Disease Control and Prevention were used in this survey. The tool is one of the built-in questionnaires in the system, namely the electronic questionnaire system of “Chinese Version of Musculoskeletal Disorders Questionnaire,” which is based on the Nordic Musculoskeletal Questionnaire. The survey content includes:(1) general demographic characteristics, including Gender, age, education level, marital status, living habits, and work of this job age, etc.; (2) Disease of WMSDs in different body parts; (3) Work Composition, including type of work, labor organization, working posture, etc. It has good reliability and validity and can be used among the Chinese occupational population (17).

### Musculoskeletal injury criteria

The US National Institute for Occupational Safety and Health criteria for musculoskeletal injuries are pain, stiffness, burning, numbness or tingling, and other uncomfortable symptoms, and at the same time satisfy (i) discomfort within the past 1 year; (ii) began to feel discomfort after engaging in the current job; (iii) no previous accident or sudden injury (affecting the local area of discomfort); and (iv) if discomfort occurs every month or lasts for more than 1 week, it is referred to a musculoskeletal disorder in this area (18).

### Data analysis

Chi-square test and binary logistic regression statistical analysis were performed using SPSS 25.0. First, differences in the prevalence of wrist pain and injury under various conditions were compared, and logistic analysis was carried out on jobs with high prevalence in animal husbandry, toy manufacturing, biopharmaceutical manufacturing, and other industries. Odds ratio (OR) values were calculated, and various types of work were compared. Descriptive analysis was performed for dependent and independent variables, and a binary logistic regression model was used to determine the statistical association between different predictors and outcome variables. We adjusted for factors, such as education level, industry, monthly income, marital status, and occupation. The models included sex, age, physical activity, and various wrist positions.

## Results

A total of 57,501 individuals were surveyed after obtaining informed consent, most of whom were males (64.8%). Height was 167.03 ± 11.57 cm, weight was 64.1 ± 16.04 kg, age was 32.8 ± 9.1 years old, this category of service was 5.75 ± 6.14 years, and total length of service was 7.51 ± 7.18 years. A majority (92.3%) of the participants were right-handed, and there was no difference in wrist injury rate between the left and right hands (*p* = 0.15). Workers with high school and technical secondary school education accounted for the largest proportion (38.1%). Workers with working age of fewer than 5 years were the most common (61.6%), and there was a difference in the prevalence of wrist injuries between ≤5 and >10 years. With an increase in working years, the prevalence of wrist injuries also increased gradually. Most of the surveyed individuals were married (61.5%), and there was no difference in the injury rate between different marital statuses. In terms of monthly income, the proportion of people with a monthly income of 3,001–5,000 Yuan was the largest, and the wrist injury rate of different income groups was similar. Differences were observed between the groups (Table 1). Because the frequency of exercise increased, the prevalence of wrist injuries gradually decreased.

**Table 1:** Wrist injury rate under different characteristics.

**Variable**	**Category**	**Number**	**Constituent**	**Prevalence of hand**	***P*-value**
			**Ratio (%)**	**injury (%)**	
Gender	Female	37,240	64.8	13	0.15
	Male	20,261	35.2	13.4	
strong hand	Right hand	53,095	92.3	13	<0.01
	Left hand	4,406	7.7	14.9	
Education	Junior high and below	15,369	26.7	14.1	<0.01
	High school and technical secondary school	21,900	38.1	14.3	
	Junior college	12,026	20.9	11.7	
	Bachelor and master degree or above	8,206	14.3	10.1	
Working age	≤5	35,432	61.6	13.4	0.03
	>5	22,096	38.4	12.8	
Smoking	No	36,530	63.5	13.1	0.01
	Occasionally	10,111	17.6	12.4	
	Frequently	9,903	17.2	14.1	
	Rimonabant	957	1.7	13	
Marriage	Spinsterhood	20,997	36.5	13.5	0.05
	Married	35,343	61.5	12.9	
	Married but living alone	1,161	2	14.8	
Monthly income	<3,000 rmb	11,220	19.5	16.8	<0.01
	3,001–5,000 rmb	28,371	49.3	13.6	
	>5,000 rmb	17,910	31.1	10	
Sporting	No	17,945	31.2	14.4	<0.01
	Occasionally	30,175	52.5	13	
	Frequently	9,381	16.3	11.1	

### Industry sickness

The survey covered 15 industries: animal husbandry, ship building and related equipment manufacturing, electronic equipment manufacturing, furniture manufacturing, construction, coal mine washing industry, civil aviation flight attendants, automobile 4S shops, automobile manufacturing, biomedical manufacturing, petrochemical industry, petrochemical industry, vegetable greenhouses, toy manufacturing, medical staff, and shoe manufacturing (*p* < 0.001). The wrist injury rates were not similar in the different industries. Five industries with the most wrist injuries were toy manufacturing, animal husbandry, automobile manufacturing, and shoe manufacturing, and the biological drug manufacturing and wrist injury rates were 29.1, 19.1, 14.9, 14.9, and 14%, respectively. Pairwise comparisons revealed differences in the prevalence of wrist injuries among the construction, automobile, toy, medical, and shoe industries (Table 2).

**Table 2:** Wrist injury rate under different industry.

**Industry**	**Number**	**Constituent**	**Prevalence of hand**	***P*-value**
		**ratio(%)**	**injury (%)**	
Animal husbandry	246	0.4	19.1	
Shipbuilding and related equipment	3,488	6.1	13	<0.01
Electronic equipment manufacturing	8,116	14.1	11	
Furniture manufacturing	4,471	7.8	12.4	
Construction industry	1,379	2.4	6.5	
Coal mining and cleaning	1,500	2.6	11.2	
Flight attendants	1,356	2.4	7.2	
4S automobile store	544	0.9	9.2	
Automobile manufacturing	21,560	37.5	14.9	
Biopharmaceutical manufacturing	243	0.4	14	
Petrochemical industry	150	0.3	4.7	
Vegetable greenhouse	243	0.4	6.6	
Toy manufacturing	333	0.6	29.1	
Medical staff	6,766	11.8	11.6	
Footwear industry	7,106	12.4	14.9	

Grouping industries by length of service and gender showed that in the toy manufacturing industry with the highest prevalence, women with more than 5 years of service had a lower prevalence of wrist injury than those with <5 years of service (Table 3).

**Table 3:** Prevalence of wrist injuries by industry by age of service and sex.

**Industry**	**Wrist injury**			
	**Working age** ≤**5**		**Working age** >**5**	
	**Male**	**Female**	**Male**	**Female**
Animal husbandry	56 (17.9)	35 (20.0)	102 (21.6)	53 (15.1)
Shipbuilding and related equipment	1,026 (11.1)	317 (16.1)	1,857 (12.9)	288 (16.7)
Electronic equipment manufacturing	3,155 (7.7)	2,844 (12.9)	761 (8.8)	1,396 (15.5)
Furniture manufacturing	2,897 (13.0)	1,177 (11.3)	289 (11.4)	108 (11.1)
Construction industry	542 (7.9)	78 (5.1)	717 (5.4)	42 (7.1)
Coal mining and cleaning	567 (11.5)	24 (0.0)	871 (11.4)	38 (10.5)
Flight attendants	191 (2.1)	612 (8.2)	109 (3.7)	444 (9.0)
4S automobile store	220 (6.4)	9 (11.1)	309 (11.0)	6 (16.7)
Automobile manufacturing	13,356 (16.3)	940 (15.2)	6,712 (12.0)	552 (14.9)
Biopharmaceutical manufacturing	72 (13.9)	58 (13.8)	37 (18.9)	76 (11.8)
Petrochemical industry	37 (0.0)	1 (0.0)	106 (5.7)	6 (16.7)
Vegetable greenhouse	10 (0.0)	16 (6.3)	144 (4.2)	73 (12.3)
Toy manufacturing	91 (35.2)	168 (28.6)	28 (28.6)	46 (19.6)
Medical staff	329 (6.7)	2,527 (9.8)	475 (9.7)	3,435 (13.6)
Footwear industry	1,423 (12.9)	11,500 (12.6)	791 (15.9)	2,198 (16.5)

We performed a univariate logistic regression analysis of occupations in 11 industries to compare the prevalence among occupations. Jobs with the highest prevalence were glue-laying (63.0%), slaughter (43.6%), car-combing (32.5%), solder (31.3%), and bracket (26.5%) workers (Table 3). The jobs in each industry were car combers in the toy manufacturing industry, slaughter workers in the animal husbandry industry, polishers in the automobile manufacturing industry, gluing workers in the shoe manufacturing industry, bottling workers in the biopharmaceutical manufacturing industry, ship building and related manufacturing industries, sanders and welders in the electronic equipment manufacturing industry, punchers in the construction industry, bracket workers in the coal mining industry, doctors in the medical industry, and furniture industry operators in the furniture manufacturing industry.

The number of people surveyed in the toy manufacturing industry was 333, and the ORs of wrist injury was 58.85 and 16.83 for rubber enamel and car comb workers, respectively. The total number of people surveyed in the livestock industry was 246, and the prevalence of milking workers was 4.3%, while the ORs of wrist injury was 3.93 times higher for feed workers and 17 times higher for slaughter workers. The number of people surveyed in the automobile manufacturing industry was 21,560, and the number of forklift workers was 2,860 with a wrist injury prevalence rate of 10.9%. The OR of wrist injury in glue coating workers was 0.73 times that of forklift workers. The ORs of illness were 1.26, 2.23, 1.63, and 1.93 for forklift workers. The number of people surveyed in the footwear industry was 7,106. The ORs of wrist injury was 1.67, 2.0 times higher for tailors and molding workers, and 2.07 times higher for gluing workers. The wrist injury rate of QC workers in biopharmaceutical manufacturing was 9.6%, and the OR of illness was 3.12 times higher for bottling workers than for QC workers (Table 4).

**Table 4:** Results of binary logistic regression analysis of different types of work in various industries.

**Type of work**	**Number**	**Number of cases (%)**	***P*-value**	**OR (95%CI)**
**Animal husbandry**				
Milker	69	3 (4.3)		
Feeder	33	5 (15.2)	0.07	3.93 (0.88, 17.57)
Butcher	39	17 (43.6)	<0.01	17 (4.55, 63.56)
**Shipbuilding and related equipment**				
Coppersmith	164	12 (7.3)		
Craneman	339	43 (12.7)	0.07	1.84 (0.94, 3.59)
Electric welder	595	91 (15.3)	0.01	2.29 (1.22, 4.29)
Ship-fitter	824	126 (15.3)	0.01	2.29 (1.165, 2.58)
Polishing operator	202	52 (25.7)	<0.01	4.39 (2.25, 8.56)
**Electronic equipment manufacturing**				
Electric welder	223	16 (7.2)		
Packer	277	36 (13.0)	0.04	1.93 (1.04, 3.58)
Fitter	1,520	276 (18.2)	<0.01	2.87 (1.70, 4.85)
Automatic operator	105	21 (20.0)	<0.01	3.23 (1.61, 6.50)
Solder work	96	30 (31.3)	<0.01	5.88 (3.02, 4.45)
**Construction industry**				
Bar placer	206	12 (5.8)		
Woodworking	324	39 (12.0)	0.02	2.21 (1.13, 4.33)
Punching workers	19	3 (15.8)	0.11	3.03 (0.78, 11.86)
**Coal mining and cleaning industry**				
Drainage workers	55	2 (3.6)		
Punching workers	100	9 (9.0)	0.23	2.62 (0.55, 12.59)
Driver	324	48 (14.8)	0.04	4.61 (1.09, 19.54)
Timberer	34	9 (26.5)	0.01	9.54 (1.92, 47.45)
**Automobile manufacturing**				
Forklift worker	2,860	313 (10.9)		
Gluer	3,726	306 (8.2)	<0.01	0.73 (0.62, 0.86)
Auto Spray-Painter	909	122 (13.4)	<0.01	1.26 (1.01, 1.58)
Electric welder	2,658	443 (16.7)	<0.01	1.63 (1.39, 1.9)
Fitter	8,275	1,589 (19.2)	<0.01	1.93 (1.70, 2.20)
Polishing operator	298	64 (21.5)	<0.01	2.23 (1.65, 3.01)
**Biopharmaceutical manufacturing**				
Quality worker	114	11 (9.6)		
Formula work	50	9 (18.0)	0.14	2.06 (0.79, 5.33)
Filling work	28	7 (25.0)	0.04	3.12 (1.08, 8.98)
**Toy manufacturing**				
Packer	71	2 (2.8)		
Comber	114	37 (32.5)	<0.01	16.83 (3.93, 72.16)
Glue maker	46	29 (63.0)	<0.01	58.85 (12.77, 271.25)
**Medical staff**				
Carer	384	29 (7.6)		
Nurse	4,057	465 (11.5)	0.02	1.593 (1.07, 2.34)
Doctor	1,782	239 (13.4)	<0.01	1.9 (1.27, 2.84)
**Footwear industry**				
Bottom-making	204	21 (10.3)		
Cropper	1,250	233 (18.6)	<0.01	1.67 (1.02, 2.75)
Shapers	634	102 (16.1)	<0.01	2.00 (1.24, 3.21)
Gluer	406	78 (19.2)	<0.01	2.07 (1.24, 3.47)
**Furniture manufacturing**				
Sanders	234	26 (11.1)		
Operator	447	80 (17.9)	0.02	1.74 (1.09, 2.80)
**4S automobile store**				
Reworkers	110	7 (6.4)		
Mechanic	235	27 (11.5)	0.142	1.91 (0.81, 4.53)
Painter	124	11 (8.9)	0.474	1.43 (0.54, 3.83)
**Flight attendants**				
Flight attendants	1,104	96 (8.7)		
Purser	73	2 (2.7)	0.093	0.30 (0.07, 1.23)
**Petrochemical industry**				
Plumber	42	2 (4.8)		
Cranes	13	1 (7.7)	0.687	1.67 (0.14, 20.01)
Welders	25	2 (8.0)	0.592	1.74 (0.23, 13.19)
**Vegetable greenhouse**				
Greenhouse worker	243	16 (6.6)		

### Logistic regression analysis

After grouping according to the length of service and sex, we analyzed the logistic regression analysis of wrist posture and found that the wrist often bent upwards and downwards, needs to be pinched with hands, grasps some objects tightly, and women with more than 5 years of service above the shoulder are at the highest OR. Placing the hand on the edge of hard and angular objects (such as the edge of a table) was highest among women with <5 years of service. Prolonged wrist flexion had the highest relationship among men within 5 years of service (Table 5).

**Table 5:** Logistic regression was used to evaluate the effect of wrist posture on wrist injury by gender and length of service.

**Variable**	**Wrist Injury OR (95% CI)** ^*^			
	**Working age** ≤**5**		**Working age** >**5**	
	**Male**	**Female**	**Male**	**Female**
Whether the wrist is often bent up/down at work	1.90 (1.69, 2.15)	1.92 (1.62, 2.27)	2.22 (1.87, 2.64)	1.89 (1.58, 2.25)
Whether the wrist is often placed on the edge of a hard, angular object	1.67 (1.54, 1.82)	1.43 (1.26, 1.62)	1.50 (1.32, 1.70)	1.40 (1.23, 1.61)
Do you need to pinch/grasp objects/tools with your hands during work	1.71 (1.46, 2.00)	1.62 (1.33, 1.98)	1.89 (1.53, 2.32)	1.68 (1.36, 2.06)
Above shoulder level	1.14 (1.03, 1.25)	1.18 (1.01, 1.39)	1.19 (1.03, 1.36)	0.92 (0.77.1.10)
Whether the wrist needs to be bent for a long time	1.89 (1.73, 2.07)	2.07 (1.80, 2.37)	1.8 (1.57, 2.06)	1.72 (1.48, 2.00)

* Is the OR value after adjusting smoking, industry, occupation.

After adjusting for education level, monthly income, marital status, dominant hand, type of work, and industry, multivariate logistic analysis showed that the OR of wrist injury in women was higher than that in men (OR = 1.24). The OR of wrist injury also increases gradually. Exercise is an associated factor against injury, and the more frequent the exercise, the greater the protection. We found that wrist posture was a dominant factor with the greatest OR value for long-term flexion of the wrist (OR = 1.86; 95% CI, 1.75–1.97), followed by wrist posture requiring frequent upward and downward flexion during work for workers' OR of wrist injury was very high (OR = 1.81; 95% CI, 1.84–2.11), which also increased the OR of wrist injury when workers needed to pinch/grab at work (OR = 1.72; 95% CI, 1.57–1.89). The OR of wrist injury was also increased (OR = 1.52; 95% CI, 1.44–1.61) when the hand was frequently placed on the edge of hard and angled objects (such as the edge of a table). People whose wrists were higher than the shoulders had a higher OR of wrist injury than those whose shoulders were below (OR = 1.11; 95% CI, 1.04–19) (Table 6).

**Table 6:** Results of binary logistic regression analysis of associated factors of wrist injury.

**Variable**	**Category**	**Number**		**Injury OR (95% CI)**	
		**No injury**	**With injury**	**Crude**	Adjusted^*^
Gender	Female	17,544	2,717		
	Male	32,404	4,836	1.07 (1.01, 1.12)	1.28 (1.18, 1.39)
Working age	≤5	30,693	4,739		
	>5	19,255	2,714	0.99 (0.94, 1.04)	1.09 (1.03, 1.15)
Sporting	No	15,361	2,584		
	Occasionally	26,249	3,926	0.94 (0.88, 0.99)	0.92 (0.87, 0.98)
	Frequently	8,338	1,043	0.83 (0.77, 0.90)	0.86 (0.80, 0.94)
Whether the wrist is often bent up/down at work	No	19,298	1,074		
	Yes	30,650	6,479	2.01 (1.87, 2.17)	1.97 (1.82, 2.12)
Whether the wrist is often placed on the edge of a hard,	No	33,997	3,478		
angular object	Yes	15,951	4,075	1.47 (1.40, 1.56)	1.54 (1.45, 1.62)
Do you need to pinch/grasp objects/tools with your hands	No	12,695	625		
during work	Yes	37,289	6,928	1.86 (1.70, 2.04)	1.73 (1.58, 1.90)
Hand position at work	Shoulder or below shoulder level	41,295	6,090		
	Above shoulder level	8,653	1,463	1.15 (1.08, 1.22)	1.16 (1.05, 1.19)
Whether the wrist needs to be bent for a long time	No	29,919	2,356		
	Yes	20,029	5,197	1.93 (1.82, 2.04)	1.87 (1.76, 2.00)

* Is the OR value after adjusting smoking, industry, occupation.

## Discussion

This study investigated the population of key industries in China to understand the prevalence and distribution of wrist injuries and to explore related epidemiological characteristics. Wrist injury is a common injury of the upper extremities. Our study found that female sex, working age, and poor wrist posture all increased the ORs of wrist injury. The ORs of some types of work is much higher than that of other work types in the same industry. The strengths of this study include the large sample size, specific case criteria, and detailed exposure to wrist posture, representing a broad range of industries, trade, and locations, adding to the generalizability of the findings.

Poor working posture can cause excessive muscle load on workers and cause the body to produce a physiological stress response. Under such stress conditions for a long time, it causes injury to muscles, nerves, and tendons. The results of this study show that among the 15 key related industries, toy manufacturing, animal husbandry, automobile manufacturing, shoe manufacturing, and biopharmaceutical manufacturing are the five industries with the greatest ORs of wrist injury. The prevalence rates of workers, including glue workers, solder workers, canners, and doctors are also higher than those of other types of workers in the same industry. The degree of mechanization in the toy manufacturing and production processes is not high. In monotonous and repetitive manual assembly line operation, the wrist requires frequent movements to complete the task (19). During work, they needed to bend over and turn around, keep their neck down for a long time, hold the tool in both hands, straighten their right hand, and keep their arms higher than their shoulders. This working method is more prone to wrist injuries than other jobs (20). In animal husbandry, for dairy farms with larger herds, the production cost is more inclined to milking in the milking room, which is equipped with modern milking equipment; therefore, the wrist injury rate is higher than that of traditional milkers. Feeders and slaughter workers still rely on manual operations to a certain extent; therefore, the ORs of musculoskeletal diseases is much greater than that of milkers. Workers in large livestock industries are exposed to factors, such as awkward postures, repetitive movements, high muscle loads, few opportunities for rest, and poor environmental conditions, which may increase the risk of WMSDs (21). Auto-manufacturing workers often need to carry heavy objects, such as auto parts and auxiliary tools for carrying out lifting operations, which will increase load on the wrist. Most auto-manufacturing workers perform assembly work, and the work content is similar every day. The body parts involved in the work of workers are impacted by the same load year after year. The prevalence rate of wrist injuries in country's footwear industry was 14.9%. An Indian survey of handmade shoes found that the prevalence of wrist pain was as high as 88.5%, and many factories in India make such handmade shoes. Sole cutting is a time-consuming, ineffective, and a physically labor-intensive job that requires manual operation and necessary skills and practice. Awkward postures, such as prolonged sitting and forward bending of the body increased the risk of WMSDs (22).

There are several notable findings regarding the association of personal and ergonomically associated factors with wrist injuries. First, our study found that the adjusted prevalence of wrist injuries differs from that of men among female workers, and that increasing working age also increased the ORs of wrist injuries, while physical activity reduces the ORs of wrist injuries. Studies have shown that the prevalence of WMSDs increases with the number of years of work, and there are differences between males and females (23). Differences in the health status of male and female workers may be due to differences in the exposure to different associated factors. Because of gender segregation in the labor market, men and women tend to work in different jobs; therefore, they face different ORs. Furthermore, even though men and women have the same job, they may have different specific tasks, which may lead to different risk exposures (23, 24). Kihlberg and Hagberg demonstrated an OR of 1.4 for wrist pain with age (25), and Davatchi et al. showed that the prevalence of wrist pain in women was 14.7%, which was higher than that in men (5.6 %) (26). We found that the prevalence of wrist injuries increased with working age, but the difference was not statistically significant. One possible reason is the healthy worker selection effect; that is, healthy workers, even in physically demanding jobs, can also maintain longer working hours. Another possibility is that pain elsewhere is more prevalent and may occur earlier than pain in the distal upper extremities. Neck and shoulder pain may prevent workers from continuing to sew, while distal upper extremity pain may take longer to develop (27). This study also identified a significant association between exercise and lower prevalence of wrist injuries. Workers who exercised were less likely to develop wrist injuries than those who did not exercise, and the more frequent the exercise, the lower the OR of injury. Exercise can provide workers with an opportunity to break free from work to restore and strengthen their bodies, while also providing mental relaxation from the high psychosocial demands of work. These effects may help improve health and reduce the risk of musculoskeletal symptoms (28).

Poor working posture is an important factor for wrist injury. Regarding the effect of wrist posture on wrist injuries found at work, the wrist is often flexed up or down, the wrist is placed on the edge of a hard object, the need to hold objects tightly, the wrist is above shoulder level, and prolonged flexion increases the OR of wrist injury. All work requires the use of the wrist, and some work also requires carrying and using tools, particularly vibrating tools. When the wrist is often bent up or down, it also increases the OR of wrist injury, and our study cannot reflect the effect of angular velocity of the wrist on wrist injury, but only the frequency of wrist movement. However, studies have shown that the risk of carpal tunnel syndrome (CTS) increases with increasing levels of wrist angular velocity (29). Many of our operations are performed on the work surface, and the wrist is often placed on the edge of hard and angular objects (such as the edge of a table), which increases the OR of wrist bumps and can easily lead to inflammation and fibers in the muscle tissue, resulting in wrist injury. The wrist tissue is also in a tense state when the hand needs to frequently pinch or grip objects or tools. Few studies have examined the position of the wrist across the body and above or below the shoulder. We found that maintaining a high working position for long periods also increased the OR of wrist injury and pain. This may be because the wrist is in a state of lifting above the shoulder, lacking certain support, and the pressure on the muscles and bones is high, resulting in injury. When the wrist is in a flexed state, the wrist muscles are in a tense state, and symptom, such as pain, is prone to occur when this state is maintained for a long time. One study also showed that when the wrist is extended more than 33° or flexed more than 49°, there is an increased risk of CTS (30). Studies have found a significant association between wrist injuries and lifting unsupported weights in each hand, grasping objects with force, and grasping objects with flexed wrists. There was also a significant association between wrist injuries and the use of hand tools, such as impact tools (e.g., jack and chisel hammers), impact wrenches, and chainsaws (31). Chronic flexion of the wrist, gripping objects with hands, upper limb or hand exertion, repetitive manipulations multiple times per minute (32), and the use of vibrating tools have been found to be associated factors for wrist pain. However, one study showed no significant increase in the frequency of CTS when the wrist repetition rate was considered independent of strength. In contrast, forceful hand repetition frequency (a measure of both exposure to forceful and repetitive hand force) was significantly associated with an increased OR of CTS, which is similar to the increased OR of wrist injury when we exert force, such as pinching objects with our hands, which shows that if there is no force, even if the wrist is often bent, it will not increase the injury. Therefore, our definition of posture has included strength because these postures require strength to complete the work, and the study also found that the prevalence of CTS appears to increase linearly with the number of hard hand repetitions up to 30/min (33).

Current research suggests that high repetition, force, awkward posture, and other sports can damage the musculoskeletal system and peripheral nerves (34, 35). A high value is closely related. Therefore, we recommend that individuals pay attention to adjusting their posture at work to prevent pain and achieve the purpose of skeletal muscle rest through reasonable work time allocation, reasonable physical exercise, warm-up, etc. to reduce the occurrence of injury.

## Limitations

This study has several limitations. First, it was not possible to make causal inferences between risk factors and WMSDs due to the cross-sectional nature of the study. Because this study used a questionnaire, the resulting report and recall biases may have affected the results, and the number of years of work used in assessing the effect of length of service on wrist injuries only includes total years in current employment and not previous employment. Therefore, to make the research more in-depth, the survey industry can be expanded and cohort and intervention studies can be conducted.

## Conclusions

Through cross-sectional surveys of 15 key industries in seven regions of China, we learned about the prevalence of wrist injuries in China's occupational population and related factors. This study provides a reference for the development of relevant measures to prevent and control the occupational population of WMSD. Toy manufacturing, automobile manufacturing, animal husbandry, and shoemaking have the highest ORs of wrist injury. Vinyl, slaughterers, car combers, solder workers, and bracket workers have the highest prevalence rates of wrist injury; therefore, there is a need to pay attention to them. Additionally, female sex and length of service increase the ORs of wrist injury, and abnormal posture of the wrist is the main cause of wrist injury. Physical exercise can reduce the OR of wrist injury, suggesting that the OR of wrist injury can be improved by adjusting working posture and performing reasonable physical exercise. Simultaneously, managers and workers in various industries need to raise health awareness, improve working conditions in a reasonable way, and protect their own health.

## Data availability statement

The original contributions presented in the study are included in the article/supplementary material, further inquiries can be directed to the corresponding authors.

## Ethics statement

The studies involving human participants were reviewed and approved by Medical Ethical Review Committee National Institute for Occupation Health and Poison Control Chinese Center for Disease Control and Prevention. The patients/participants provided their written informed consent to participate in this study. Written informed consent was obtained from the individual(s) for the publication of any potentially identifiable images or data included in this article.

## Author contributions

NC and GuL: article writing. XS, MZ, HuZ, RL, YiL, GaL, ZR, YY, HS, HeZ, JLi, BQ, DW, QZ, ZL, RW, JC, DZ, LM, YoL, JLiu, CZ, TL, and ZW: data handling. QC and NJ: provide topic selection and writing guidance. All authors contributed to the article and approved the submitted version.

## Funding

This study was funded by the Project of Occupational Health Risk Assessment and the National Occupational Health Standard Formulation of the National Institute of Occupational Health and Poison Control (Project No. 131031109000160004) and National Key R&D Program - Research on Key Technologies and Intervention Strategies for the Prevention and Control of Work-related Diseases and Occupational Injuries (2022YFC2503205).

## Conflict of interest

The authors declare that the research was conducted in the absence of any commercial or financial relationships that could be construed as a potential conflict of interest.

## Publisher's note

All claims expressed in this article are solely those of the authors and do not necessarily represent those of their affiliated organizations, or those of the publisher, the editors and the reviewers. Any product that may be evaluated in this article, or claim that may be made by its manufacturer, is not guaranteed or endorsed by the publisher.
